# Simple Rain-Shelter Cultivation Prolongs Accumulation Period of Anthocyanins in Wine Grape Berries

**DOI:** 10.3390/molecules190914843

**Published:** 2014-09-17

**Authors:** Xiao-Xi Li, Fei He, Jun Wang, Zheng Li, Qiu-Hong Pan

**Affiliations:** 1 Center for Viticulture and Enology, College of Food Science and Nutritional Engineering, China Agricultural University, Beijing 100083, China; E-Mails: lixxcau@gmail.com (X.-X.L.); wheyfey@cau.edu.cn (F.H.); junwang1966@163.com (J.W.); 2 Food Science and Human Nutrition Department, Institute of Food and Agricultural Sciences, University of Florida, Gainesville, FL 32611, USA; E-Mail: Jameslee0221@ufl.edu

**Keywords:** anthocyanins, wine grape, rain-shelter cultivation, open-field cultivation

## Abstract

Simple rain-shelter cultivation is normally applied during the grape growth season in continental monsoon climates aiming to reduce the occurrence of diseases caused by excessive rainfall. However, whether or not this cultivation practice affects the composition and concentration of phenolic compounds in wine grapes remains unclear. The objective of this study was to investigate the effect of rain-shelter cultivation on the accumulation of anthocyanins in wine grapes (*Vitis vinifera* L. Cabernet Sauvignon) grown in eastern China. The results showed that rain-shelter cultivation, compared with the open-field, extended the period of rapid accumulation of sugar, increased the soluble solid content in the grape berries, and delayed the senescence of the green leaves at harvest. The concentrations of most anthocyanins were significantly enhanced in the rain-shelter cultivated grapes, and their content increases were closely correlated with the accumulation of sugar. However, the compositions of anthocyanins in the berries were not altered. Correspondingly, the expressions of *VvF3'H*, *VvF3'5'H*, and *VvUFGT* were greatly up-regulated and this rising trend appeared to continue until berry maturation. These results suggested that rain-shelter cultivation might help to improve the quality of wine grape berries by prolonging the life of functional leaves and hence increasing the assimilation products.

## 1. Introduction

The Chinese wine industry has developed with unprecedented speed in recent years in terms of both production and consumption. Currently, it is one of the top 10 wine-producing countries in the world regarding area under vine and wine volume produced. The national appetite for wine has correspondingly more than doubled in the past two decades [1]. China’s wine regions spread across the breadth of the country. On the humid and monsoonal east coast, the provinces of Shandong and Hebei and Tianjin City are responsible for a large amount of China’s national production. These areas have formed the characteristic wine industry clusters. The terroir of Shandong Province avoids the harsh continental extremes of the center of China, and instead has a maritime climate with cooler summers and warmer winters. The Shandong wine-producing regions are affected by the East Asian Monsoon, a weather system that brings cool and moist air from the Pacific Ocean to the shores of the province, causing summer rains. Fungal vine diseases caused by high rainfall are an important concern for vignerons in the late summer and early autumn.

Rain-shelter cultivation is a common kind of canopy management consisting of building a polyethylene (PE) film roof one meter from the top of the grapevine canopy. In China, rain-shelter cultivation is usually implemented in the production of table grape berries along the midstream and downstream areas of Yangtze River and in the south [2]. A large amount of viticulture practice has indicated that rain-shelter cultivation can effectively eliminate the incidence of major diseases, such as downy mildew (*Plasmoparaviticola*), powdery mildew (*Uncinulanecator*), botrytis (*Botrytiscinerea*), rip rot (*Glomerellacingulata*), and sour rot (imperfect yeasts), by keeping rainwater away from leaves and fruits. Under this circumstance, the use of pesticides could be remarkably reduced [3].

**Figure 1:**
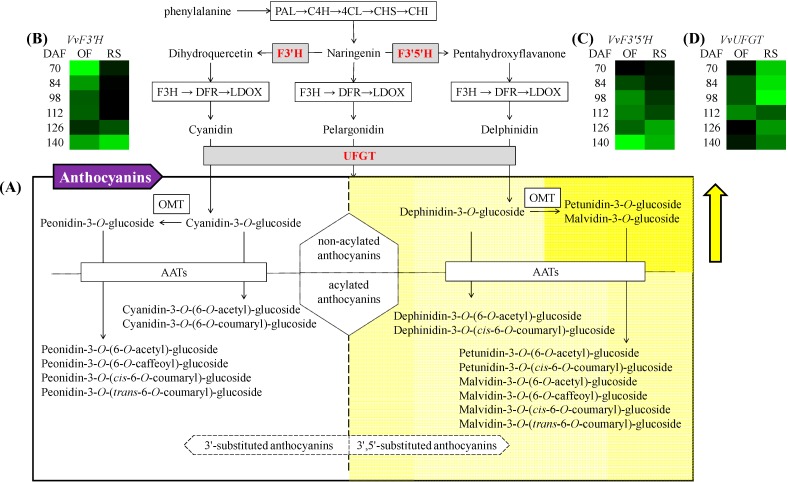
Biosynthetic pathway of anthocyanidins in grape (based on [12], only detected anthocyanins are listed in this figure, (**A**)). The relative expression amount of *VvF3'H* (**B**), *VvF3'5'H* (**C**) and *VvUFGT* (**D**) is shown using three heat maps. Green squares from dark to bright represent the gene expression levels from high to low during the mature period (DAF70 to 140) with respect to both open-field cultivation (OF) and rain-shelter cultivation (RS). The yellow square shown in the anthocyanins block indicates that the compounds from this branch have higher concentration in berry skins under rain-shelter cultivation in comparison to open-field at commercial harvest. Abbreviations: DAF, days after flowering; PAL, phenylalanine ammonia-lyase; C4H, cinnamate 4-hydroxylase; 4CL, 4-coumarate: CoA ligase; CHS, chalcone synthase; CHI, chalcone isomerase; F3'H, flavonoid 3'-hydroxylase; F3'5'H, flavonoid 3',5'-hydroxylase; F3H, flavanone 3-hydroxylase; DFR, dihydroflavonol reductase; LDOX, leucoanthocyanidin dioxygenase; UFGT, UDP-glucose: flavonoid 3-*O*-glucosyltransferase; OMT, o-methyltransferase; AATs, anthocyanin acyltransferases.

Anthocyanins are a crucial class of phenolic pigments in grapes and wine, and they contribute to the appearance (color), sensory quality (chromaticity and color tone), stability (aging potential), and potential human health benefits [4,5,6]. Anthocyanins are accumulated predominantly in the skins of grapes from the beginning of véraison. The biosynthesis of anthocyanins has two important branches, the 3'-substituted anthocyanin synthesis (for example, cyanidin-3-*O*-glucoside and peonidin-3-*O*-glucoside) led by flavonoid-3'-hydroxylase (F3'H), and the 3'5'-substituted anthocyanin synthesis (for example, delphindin-3-*O*-glucoside, petunidin-3-*O*-glucoside and malvidin-3-*O*-glucoside) regulated by flavonoid-3'5'-hydroxylase (F3'5'H) (Figure 1).

Some literature has reported that rain-shelter cultivation effectively delayed the maturation of grapes [7], slowed the sugar accumulation by reducing photosynthetically active radiation (PAR), significantly increased berry and cluster weight, and improved economic returns [8]. Although rain-shelter cultivation has been studied for its commercial value on table grapes and other fruits during the past several decades [8,9,10,11], few studies have focused on wine grapes. Some problems remain to be solved. For example, (i) does rain-shelter cultivation delay the maturity of wine grape berries and decrease their quality?; (ii) what is the effect on the composition and content of anthocyanins in the skins of grape berries under rain-shelter cultivation? This study aimed to investigate the effect of simple rain-shelter cultivation on the accumulation of anthocyanins in *Vitis vinifera* L. Cabernet Sauvignon berries, and to evaluate the prospects for application in the production of high-quality wine grapes in rainy regions.

## 2. Results and Discussion

### 2.1. Total Soluble Solid and pH Value

**Figure 2:**
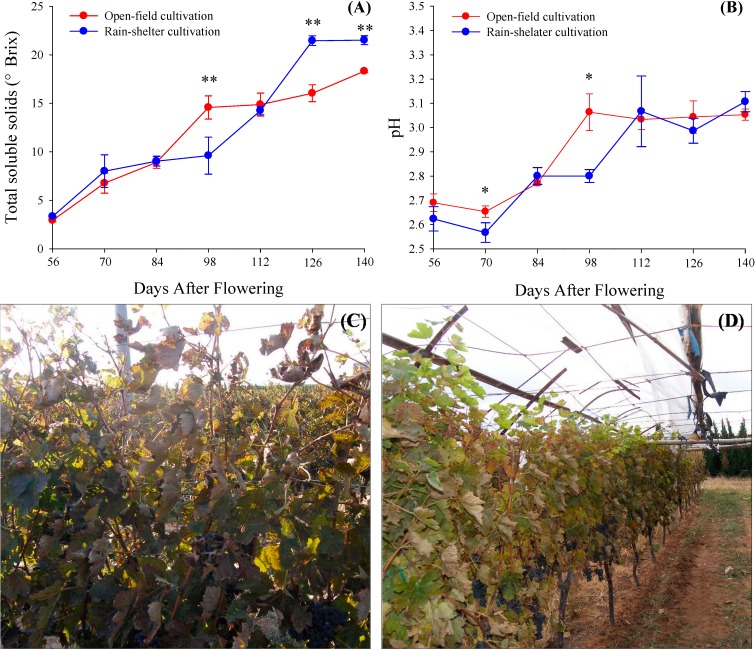
The variations of total soluble solids **(A)** and pH **(B)** of grape juice between vines grown under open-field cultivation and rain-shelter cultivation; and canopies of vines grown under open-field cultivation **(C)** and rain-shelter cultivation **(D)** at commercial harvest. Data are mean ± standard deviation. The symbol “**” or “*” above each set of columns represents that there are significant difference between rain-shelter cultivation and open-field cultivation by Duncun analysis at 0.01 level (*p* < 0.01) or 0.05 level (*p* < 0.05). *n* = 6.

Figure 2 shows the variation of total soluble solids (°Brix) and pH value in the grape berries under rain-shelter and in open-field cultivation from 2 weeks before véraison (56 DAF) to the technological harvest (126 DAF) and post-maturity (140 DAF), respectively. From 56 DAF, total soluble solids in the grape berries under the open-field cultivation rapidly accumulated by 98 DAF and then slowly increased until the end of the experiment. Correspondingly, under rain-shelter cultivation, the rapid accumulation period of soluble solids in the grape berries was extended to 126 DAF and the peak value appeared at 126 DAF. During post-maturity, total soluble solids in the berries of rain-shelter cultivation showed no significant changes. At harvest, the content of sugar in the berries under rain-shelter cultivation was significantly higher than the control, reaching 21.5 °Brix compared with 16.0 °Brix in the open-field cultivated berries. Similarly, the maximum pH values were observed at 98 DAF and 112 DAF, under the open-field cultivation and rain-shelter cultivation respectively. After the end of véraison, pH values in the berries showed no significant differences between the two cultivation modes.

The longer and greater accumulation of sugar in the berries under rain-shelter cultivation was possibly related to a delay in canopy leaf senescence. We observed that the grapevines under the rain-shelter cultivation still had more green leaves, even at harvest, in comparison with the vines in the open-field cultivation (Figure 2C,D). This indicates that the rain-shelter cultivation could effectively prolong the life of functional leaves and therefore enhance the production ability of photosynthetic assimilates of whole vines. The visible prolonging of functional leaves is inferred to be related to a lower ultraviolet radiation (UVR) and lower canopy temperature inside the rain-shelter. Simple rain-shelter cultivation improves the quality of a great number of horticultural products, such as tomato, pepper, oat, blueberry, loquat and table grape [10,13,14,15,16,17]. Previous research indicated that the plastic covering materials used in rain-shelter cultivation can filter out part of UVR and PAR, and help avoid the direct sunlight at noon [7,10]. In a field, temperature above the top of canopy was about 5°C higher than inside the canopy [18], and the photosynthesis in grape leaves was inhibited by UVR, specifically by UV-B radiation (UVBR) [19]. Similar inhibitory effects also had been observed in other plant tissues. For example, high irradiance on bayberry leaves resulted in photo-inhibition and photo-damage by inactivation of photosystem II reaction centers [12]. These results obtained in the previous studies all imply that high irradiation can accelerate leaf senescence. Accordingly, it is considered that rain-shelter cultivation can effectively delay the process of leaf senescence and maintain the ability of leaf photosynthesis.

### 2.2. Accumulation of Anthocyanins and Expression of VvUFGT

The rain-shelter cultivation did not alter the composition of anthocyanins in the grape berries (Table 1). The content of anthocyanins increased along with berry maturation period, and a total of 19 anthocyanins were identified in the berry skins at 140 DAF. The rain-shelter cultivation could advance the generation of new anthocyanins during the maturation period of the grape berry. Malvidin-3-*O*-(6-*O*-caffeoyl)-glucoside and peonidin-3-*O*-(6-*O*-caffeoyl)-glucoside were quantified in the rain-shelter cultivated grapes at 105 DAF and 112 DAF, respectively, whereas both of them were detected only at 140 DAF (post-maturity stage) in the open-field cultivated grapes.

**Figure 3:**
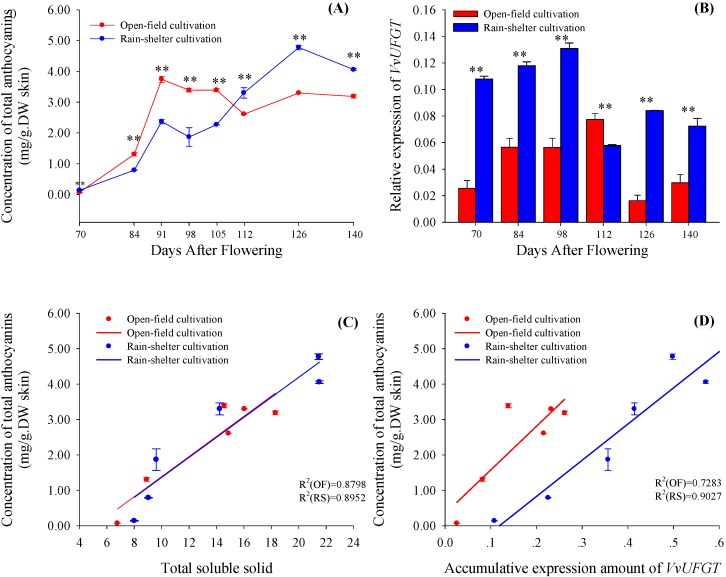
The variation of concentration of total anthocyanins (**A**) and relative expression of *VvUFGT* (**B**) in berry skins of vines grown under open-field cultivation (OF) and rain-shelter cultivation (RS). The linear correlations between evolution of total anthocyanins and total soluble solids (**C**), as well as between total anthocyanins and *VvUFGT* expression (**D**). Data are mean ± standard deviation. The symbol “**” or “*” above each set of columns represents that there are significant difference between rain-shelter cultivation and open-field cultivation by Duncun analysis at 0.01 level (*p* < 0.01) or 0.05 level (*p* < 0.05). *n* = 6.

The accumulation of anthocyanins displayed different trends in the open-field and rain-shelter cultivated grapes (Figure 3A). Under open-field cultivation conditions, the total concentration of anthocyanins in the grape skins increased to the highest value at 91 DAF (3rd week after coloring), and then decreased slightly. However, the increasing trend of the total anthocyanin content in the rain-shelter cultivated grapes was extended to 126 DAF. Interestingly, the total concentration of anthocyanins in the rain-shelter cultivated grapes was always lower than that in the open-field cultivated grapes from 70 to 105 DAF, but the contrary occurred during the following ripening phases. UDP-glucose:flavonoid 3-*O*-glucosyltransferase (UFGT) has been demonstrated to be a critical enzyme for anthocyanin biosynthesis in grape berries, and it can transfer the glucose residues of the UDP-glucose molecule to the 3 position of anthocyanidin in the C ring to form anthocyanins [20]. The expression of *VvUFGT* was significantly up-regulated in the rain-shelter cultivated grapes during the whole ripening period, with the exception of the 112 DAF (Figure 3B). The variation of the total anthocyanin concentration paralleled well that of the soluble solids in the process of berry maturation and to the accumulative expression of *VvUFGT* under either of cultivation modes (Figure 3C,D). For the rain-shelter cultivated grapes, the correlation was more significant in terms of the correlation coefficient (the R^2^ value was shown in Figure 3C,D).

**Table 1:** Content of individual anthocyanins in grape skins under rain-shelter cultivation and open-field cultivation.

Anthocyanins (mg/kg DW skin)	70 DAF		84 DAF		91 DAF		98 DAF		105 DAF		112 DAF		126 DAF		140 DAF	
	Rain-Shelter	Open-Field	Rain-Shelter	Open-Field	Rain-Shelter	Open-Field	Rain-Shelter	Open-Field	Rain-Shelter	Open-Field	Rain-Shelter	Open-Field	Rain-Shelter	Open-Field	Rain-Shelter	Open-Field
Dephinidin-3-*O*-glucoside	14.5 ± 0.4a	7.8 ± 0.9b	77.6 ± 0.9b	172.3 ± 5.5a	212.4 ± 7.2b	420.8 ± 18.6a	94.8 ± 13.2b	238.4 ± 7.2a	149.4 ± 3.2b	286.4 ± 8.0a	191.7 ± 4.9a	174.0 ± 4.9b	491.5 ± 26.2a	268.7 ± 11.6b	403.7 ± 13.6a	181.2 ± 7.1b
Petunidin-3-*O*-glucoside	9.8 ± 0.4a	5.3 ± 0.7b	50.3 ± 3.5b	95.5 ± 3.8a	146.5 ± 11.4b	252.4 ± 8.4a	75.5 ± 12.4b	166.5 ± 11.5a	106.1 ± 9.9b	189.4 ± 5.2a	140.6 ± 5.0a	109.9 ± 2.8b	303.8 ± 17.3a	164.5 ± 7.7b	231.6 ± 1.6a	122.1 ± 10.7b
Malvidin-3-*O*-glucoside	34.8 ± 2.0a	12.9 ± 0.8b	280.9 ± 1.0b	392.6 ± 13a	895.3 ± 21.2b	1291.2 ± 32.2a	720.8 ± 103.0b	1329.1 ± 22.6a	882.9 ± 0.9b	1252.6 ± 25.7a	1366.1 ± 59.2a	998.3 ± 1.8a	1831 ± 23.2a	1291.3 ± 1.6b	1595.4 ± 1.3a	1323.3 ± 5.6b
Dephinidin-3-*O*-(6-*O*-acetyl)-glucoside	8.1 ± 0.4a	3.8 ± 0.1b	28.8 ± 1.1b	51.8 ± 1.7a	73.9 ± 0.2b	121.8 ± 0.8a	47.2 ± 2.4b	85.5 ± 3.9a	59.6 ± 0.4b	95.6 ± 0.8a	79.7 ± 3.4a	72.8 ± 1.7a	148.6 ± 0.2a	90.5 ± 4.8b	135.9 ± 3.9a	82.3 ± 0.6b
Petunidin-3-*O*-(6-*O*-acetyl)-glucoside	6.1 ± 0.6a	3.4 ± 0.0b	19.1 ± 0.2b	35.3 ± 1.8a	53.0 ± 4.1b	87.0 ± 1.7a	29.5 ± 4.2b	61.8 ± 2.5a	41.3 ± 3.0b	68.5 ± 0.1a	54.8 ± 3.7a	39.1 ± 2.3b	104.9 ± 0.9a	52.0 ± 1.5b	89.5 ± 5.3a	41.1 ± 4.2b
Malvidin-3-*O*-(6-*O*-acetyl)-glucoside	28.0 ± 3.1a	10.9 ± 0.5b	153.0 ± 8.2b	196.6 ± 6.1a	475.2 ± 2.7b	647.8 ± 10.3a	472.2 ± 4.6b	782.6 ± 3.3a	516.3 ± 13.8b	709.5 ± 0.8a	782.3 ± 48.2a	582.4 ± 3.0b	883.6 ± 1.7a	673.8 ± 2.4b	836.4 ± 10.9a	700.2 ± 10.1b
Dephinidin-3-*O*-(*cis*-6-*O*-coumaryl)-glucoside	-	-	4.9 ± 0.1b	9.4 ± 0.8a	16.6 ± 1.7b	27.3 ± 3.7a	10.2 ± 4.8b	20.4 ± 0.9a	13.8 ± 0.8b	22.8 ± 0.3a	17.4 ± 1.8a	14.6 ± 0.1a	30.4 ± 0.5a	18.5 ± 0.3b	25.6 ± 1.6a	16.0 ± 0.4b
Malvidin-3-*O*-(6-*O*-caffeoyl)-glucoside	-	-	-	-	-	-	-	-	5.3 ± 0.5	-	7.4 ± 0.8	-	7.7 ± 0.5	-	11.4 ± 1.7a	9.4 ± 0.2b
Petunidin-3-*O*-(*cis*-6-*O*-coumaryl)-glucoside	-	-	4.4 ± 0.3b	7.9 ± 0.8a	12.7 ± 0.3b	19.4 ± 0.8a	7.8 ± 1.2b	15.3 ± 0.4a	9.8 ± 0.4b	18.0 ± 0.0a	11.2 ± 1.4a	12.1 ± 0.3a	21.5 ± 0.5a	14.0 ± 0.1b	16.8 ± 1.4a	11.8 ± 0.4b
Malvidin-3-*O*-(*cis*-6-*O*-coumaryl)-glucoside	-	-	5.9 ± 0.3a	7.7 ± 1.1a	17.1 ± 1.2a	21.1 ± 1.1a	17.9 ± 2.3b	25.0 ± 0.5a	18.4 ± 0.2a	20.7 ± 0.4a	26.2 ± 3.2a	25.4 ± 1.0a	17.2 ± 1.4b	23.6 ± 1.9a	16.7 ± 0.9b	27.8 ± 1.4a
Malvidin-3-*O*-(*trans*-6-*O*-coumaryl)-glucoside	4.5 ± 0.2a	3.0 ± 0.3b	38.6 ± 4.3b	52.5 ± 5.3a	156.5 ± 1.0b	221.2 ± 6.8a	163.1 ± 29.1b	274 ± 5.3a	175.8 ± 11.3b	250.2 ± 2.7a	252.1 ± 18.4a	255.3 ± 6.5a	294.3 ± 3.3a	267.4 ± 9.9a	249.5 ± 2b	330.2 ± 9.1a
Cyanidin-3-*O*-glucoside	6.7 ± 0.2a	4.9 ± 0.5a	24.9 ± 2.2b	64.0 ± 2.4a	51.2 ± 2.2b	122.2 ± 3.9a	26.6 ± 4.1b	47.7 ± 9.1a	35.0 ± 4.9b	71.7 ± 1.1a	43.2 ± 1.5a	35.1 ± 3.4b	106.2 ± 2.9a	59.9 ± 1.5b	68.1 ± 3.6a	33.2 ± 2.9b
Peonidin-3-*O*-glucoside	13.0 ± 0.5a	7.8 ± 0.5b	61.0 ± 0.3b	129.2 ± 4.6a	148.0 ± 6.6b	299.2 ± 6.9a	108.9 ± 17.9b	188.7 ± 5.3a	140.1 ± 2.8b	225.8 ± 4.0a	178.5 ± 7.8a	160.1 ± 1.2b	316 ± 6.8a	213.7 ± 0.8b	212.3 ± 3.9a	176.0 ± 5.7b	
Cyanidin-3-*O*-(6-*O*-acetyl)-glucoside	3.5 ± 0.2a	2.6 ± 0.0a	8.0 ± 0.6b	17.8 ± 0.2a	17.7 ± 2.6b	33.3 ± 0.6a	12.7 ± 4.6a	21.6 ± 2.4a	15.9 ± 2.7b	24.9 ± 0.2a	21.3 ± 1.9a	13.5 ± 1.3b	34.5 ± 0.3a	19.6 ± 1.3b	27.1 ± 3.0a	17.0 ± 2.6b	
Peonidin-3-*O*-(6-*O*-acetyl)-glucoside	7.4 ± 1.0a	4.4 ± 0.3b	21.3 ± 0.6b	41.6 ± 1.9a	53.3 ± 1.5b	97.5 ± 0.9a	46.9 ± 0.9b	75.1 ± 0.8a	56.1 ± 0.6b	83.8 ± 0.5a	70.2 ± 4.1a	61.5 ± 1.1b	101.7 ± 0.1a	72.7 ± 0.1b	75.8 ± 2.5a	63.9 ± 1.4b	
Peonidin-3-*O*-(6-*O*-caffeoyl)-glucoside	-	-	-	-	-	-	-	-	-	-	5.5 ± 0.2	-	5.3 ± 0.3	-	5.8 ± 1.1	Trace	
Cyanidin-3-*O*-(6-*O*-coumaryl)-glucoside	-	-	-	7.7 ± 0.7	7.2 ± 0.4b	15.5 ± 0.3a	4.7 ± 0.7b	8.2 ± 0.0a	6.2 ± 0.0b	12.7 ± 1.5a	7.2 ± 0.7	6.9 ± 0.1	11.5 ± 0.4a	10.8 ± 1.3a	8.4 ± 0.8a	6.4 ± 0.2a	
Peonidin-3-*O*-(*cis*-6-*O*-coumaryl)-glucoside	-	-	2.5 ± 0.1a	4.0 ± 0.4a	4.4 ± 0.3b	7.0 ± 0.4a	4.4 ± 0.2a	5.9 ± 0.1a	5.1 ± 0.2a	6.0 ± 0.6a	6.3 ± 1.2a	5.5 ± 0.3a	5.7 ± 0.9a	6.1 ± 0.5a	5.3 ± 1.1a	5.5 ± 0.2a	
Peonidin-3-*O*-(*trans*-6-*O*-coumaryl)-glucoside	2.7 ± 0.2a	2.3 ± 0.1a	8.9 ± 1.0b	19.4 ± 2.4a	26.7 ± 0.2b	55.2 ± 3.0a	24.0 ± 4.0b	42.5 ± 0.5a	31.2 ± 1.9b	52.0 ± 0.0a	38.9 ± 4.1a	43.6 ± 0.1a	59.2 ± 1.1a	50.6 ± 2.6b	41 ± 0.3b	48.0 ± 3.3a	

Malvidin-3-*O*-glucoside was used as the external standard for quantification. Data are mean ± standard deviation. “-” represents “not detected”. Different letters in each category represent that the concentrations of this anthocyanin had significant difference between the rain-shelter cultivation and the open-field cultivation at *p* ≤ 0.05 by Duncun analysis.

These results showed that rain-shelter cultivation did not alter the compositional proportion of anthocyanins, but increased the concentrations of almost all the anthocyanin components detected in the ripening berries. This suggested that rain-shelter cultivation could promote the whole anthocyanin biosynthetic pathway rather than a specific branch. This promotion was possibly achieved by enhancing the supplementation of photo-assimilates from the grape leaves since the senescence of the leaves under the rain-shelter cultivation was delayed. Previous investigations have shown that rain-shelter cultivation contributes to a higher yield [8,9], but few of them were focused on fruit quality. For example, Meng *et al.* [2] investigated the effect of rain-shelter cultivation mode on accumulation of phenolic compounds and incidence of grape diseases, and found that both phenolic compounds of grape skins and incidence of grape diseases decreased under rain-shelter cultivation compared with open-field cultivation. They explained that lower solar radiation and higher air humidity in the rain-shelter could produce a larger influence on the anthocyanin accumulation than air temperatures. However, in the present study, the accumulation periods of sugar and anthocyanins were both prolonged in the grape berry under the rain-shelter cultivation, which might be attributed to the delay of senescence of grape leaves and the change of sink-source relationship in this cultivation mode.

The promotion in all kinds of anthocyanins was caused by both an up-regulation of *VvUFGT* expression and a continuous accumulation of sugar. In fact, the expression level of *VvUFGT* did not correspond to the production of anthocyanins in the early stage of berry maturation under the rain-shelter cultivation and the accumulation speed of anthocyanin in the rain-shelter treated berries during véraison was lower than that in the open-field berries. These results suggest that during this period anthocyanin synthesis was still restricted by the production of upstream metabolites (like sugar). This also demonstrated a close correlation between the supplement of photo-assimilates and the accumulation of anthocyanins.

### 2.3. 3'5'-Substituted and 3'-Substituted Anthocyanins

**Figure 4:**
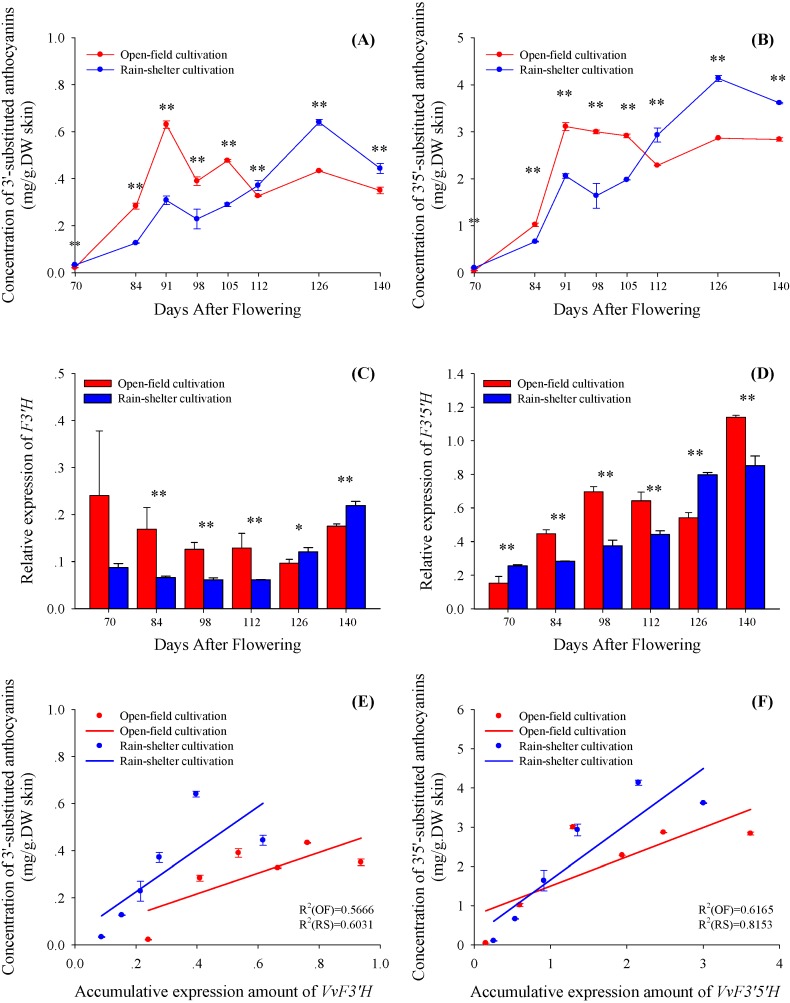
The variation of concentration of 3'-substituted anthocyanins (**A**), 3'5'-substituted anthocyanins (**B**), relative expression of *VvF3'H* (**C**) and *VvF3'5'H* (**D**) in berry skins of vines grown under open-field cultivation (OF) and rain-shelter cultivation (RS) in the process of maturing. And the correlation between content of total 3'-substituted anthocyanins and accumulative expression amount of *VvF3'H* (**E**), as well as between 3'5'-substituted anthocyanins and *VvF3'5'H* expression (**F**). Data are mean ± standard deviation. The symbol “**” or “*” above each set of columns represents that there are significant differences between rain-shelter cultivation and open-field cultivation by Duncun analysis at 0.01 level (*p* < 0.01) or 0.05 level (*p* < 0.05). *n* = 6.

In order to help to understand the influence of rain-shelter cultivation on the different branches of the anthocyanin synthetic pathway in grape berries, we grouped the detected anthocyanins into two types according to their B-ring substitutions: 3'5'-substituted anthocyanins produced from F3'5'H branch (also called delphinidin-type) and 3'-substituted anthocyanins from F3'H branch (also called cyanidin-type). Of the 19 anthocyanins identified in this study, there were eleven 3'5'-substituted anthocyanins, accounting for greater than 65% of total anthocyanin concentration, and eight 3'-substituted ones (Table 1). Like the variation of the total anthocyanin concentration, these two types of anthocyanins under rain-shelter cultivation showed lower concentrations from 70 to 105 DAF, but higher concentrations in the following maturity stages compared to those under open-field cultivation (Figure 4A,B). Moreover, the amplitude of variation of 3'-substituted anthocyanins was similar to that of 3'5'-substituted ones in post-véraison period, indicating that these were similar effects of rain-shelter cultivation on the two branch pathways of anthocyanin biosynthesis.

Generally, 3'5'-substituted and 3'-substituted anthocyanins are the downstream products of flavonoid metabolism, in which flavonoid 3'-hydroxylase (F3'H) and flavonoid 3'5'-hydroxylase (F3'5'H) lead to two branch pathways (Figure 1). The transcriptional expressions of *VvF3'H* and *VvF3'5'H* in the berry skins showed different trends under these two cultivation modes during the ripening period (Figure 4C,D). The relative expression increase of *VvF3'H* was observed in the berries under the rain-shelter cultivation. However, the open-field cultivated grape berries showed a decreasing trend on the expression of *VvF3'H* from véraison through ripening, and then was recovered at the post-maturity period. The relative expression of *VvF3'5'H* was enhanced during the ripening stages in the berries under both the open-field and rain-shelter cultivations.

It should be observed that the evolution of 3'-substituted and 3'5'-substituted anthocyanins along with berry maturation were roughly accompanied with the accumulative expression pattern of *VvF3'H* and *VvF3'5'H* under both of the cultivations, respectively (Figure 4E,F). Comparably, the relationship between the accumulation of anthocyanins and the expression of the genes tended to be closer in the berries under the rain-shelter cultivation than the open-field cultivation. These observations suggested that rain-shelter cultivation might promote the synthetic branches of both 3'5'-substitued and 3'-substitued anthocyanins in grape berries. The results indicated that the rain-shelter cultivation can promote the two branches of anthocyanin biosynthesis to a similar extent.

### 2.4. Acylating and Methylating Modification of 3'5'-Substituted and 3'-Substituted Anthocyanins

**Figure 5:**
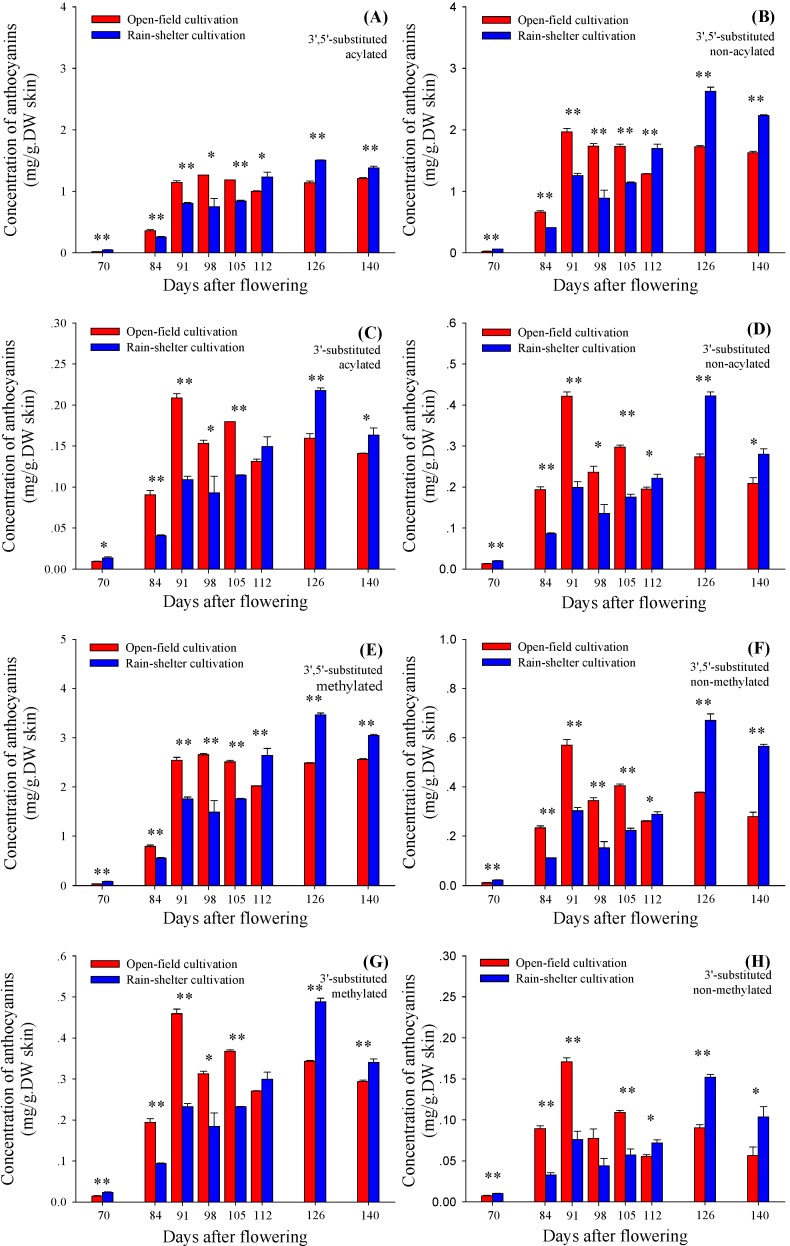
The variation in the concentrations of different kinds of anthocyanins in berry skins of vines grown under open-field cultivation and rain-shelter cultivation during berry maturation. These compounds are divided into acylated (**A**) and non-acylated (**B**) anthocyanins from F3'5'H branch (3',5'-substituted) as well as acylated (**C**) and non-acylated (**D**) anthocyanin from F3'H branch (3'-substituted). Another classification is made according to methylation in 3'- or 3',5'-position(s) of (**E**) and non-methylation (**F**) anthocyanins from F3'5'H branch, as well as methylation in 3'-position of (**G**) and non-methylation (**H**) anthocyanins from F3'H branch, respectively. Data are mean ± standard deviation. The symbol “**” or “*” above each set of columns represents that there are significant differences between rain-shelter cultivation and open-field cultivation by Duncun analysis at 0.01 level (*p* < 0.01) or 0.05 level (*p* < 0.05). *n* = 6.

Regarding the acylation on glycosyl residues of anthocyanins, the compounds can be divided into two kinds: acylated and non-acylated anthocyanins. Compared with homologous non-acylated anthocyanins, the acylated ones have a better stability in water [21]. Under these two cultivation modes, the concentration of acylated anthocyanins accounted for about 40%, most of which were acetylated. The proportion presented a decline initially, followed by an increase. Besides the acylated delphindin-type anthocyanins (3'5'-substituted), the other three kinds of anthocyanins had similar accumulation patterns (Figure 5A–D). Under the open-field cultivation, the highest concentration appeared at 91 DAF, followed by a big reducing concentration by 126 DAF. However, under rain-shelter cultivation the accumulation increased slowly and reached the highest at 126 DAF. The four kinds of anthocyanins (acylated and non-acylated cyanidin-type anthocyanins and delphindin-type anthocyanins, respectively) under the rain-shelter cultivation had lower levels than those under the open-field during 70–105 DAF, but showed significantly higher levels during the following ripening time.

Non-acylated delphindin-type anthocyanins showed the highest concentration and the greatest change, which played the primary role in leading to a higher concentration of total anthocyanins under the rain-shelter cultivation. Combined with the results in Table 1, it was found that the main compounds causing the great change of 3'5'-substitued anthocyanins under rain-shelter cultivation were malvidin-3-*O*-(6-*O*-acetyl)-glucoside and malvidin-3-*O*-glucoside.

The methylation of the B-ring on anthocyanin can bring down the chemical activity of the hydroxyl groups [22], and reduce the activity of hydroxyl groups of flavonoid substances [23]. The 3'-methylation and 3',5'-methylation of the B-ring can also increase the wavelength of anthocyanin maximum absorption. Figure 5E–H show the trends of the effect of cultivation modes on the accumulation of four kinds of anthocyanins (methylated/non-methylated of cyanidin-type/delphindin-type). These trends were similar with that of the impact on the total concentration of anthocyanins. That is, the rain-shelter cultivation reduced the accumulation of these four kinds of anthocyanins during 70–105 DAF, but enhanced the accumulation at the following ripening stages.

Acylation, especially acetylation, can significantly increase the stability of anthocyanins. The absorbing wavelength of acylated anthocyanins is blue shifted relative to the corresponding non-acylated ones, owing to the formation of fold structure inside aromatic nucleus molecules [21]. Methylated anthocyanins also provide wine with redder colorations [24]. In this study, the berries under the rain-shelter cultivation had higher concentration of acylated anthocyanins at the technological harvest and post-maturity dates. This indicated that this cultivation mode in the experimental region could help to improve the quality of grape berry and wine. In addition, the previous studies suggested that grapes under rain-shelter cultivation were suggested to be late-harvested because of the delay of ripeness and sugar accumulation [11,25]. In regions with grape growing season such as Penglai, late harvest under rain-shelter cultivation is not required.

### 2.5. Climate Characters

**Figure 6:**
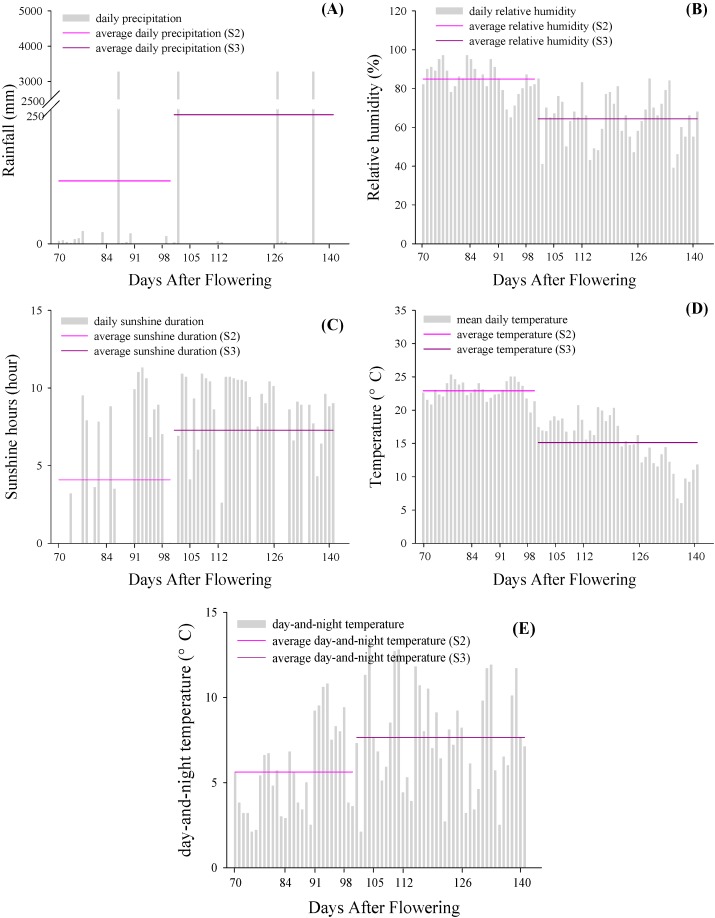
Rainfall (**A**), relative humidity (**B**), sunshine hours (**C**), mean daily temperature (**D**) and day-and-night temperature (**E**) during veraison (stage 2) and maturation period (stage 3) in 2010, Penglai region, Shandong Province, China.

The meteorological data of 2010 in Penglai region is shown in Figure 6. During véraison (70–98 DAF), the average relative humidity was above 80% (Figure 6B), while the high temperature appeared at the same period (Figure 6D). There were four heavy rain episodes during véraison, at the end of véraison, at 127 DAF, and at the post-maturity stage. Rainfall was accompanied by an increase of air humidity, a decrease of diurnal temperature, and a lack of sunshine. The daily average temperature declined gradually, from above 25 °C to around 10 °C. Taken together, there were two brief periods during time series from véraison through ripening, DAF 91 to 98 and 112 to 126, which were similar with “drier with cool-warm climate”, which contributed to the promotion of anthocyanin concentration, with higher temperature and sunshine hours, and lower rainfall and relative humidity [26].

## 3. Experimental Section

### 3.1. Field Treatment and Sample Collection

The field work was conducted during the grape growth season of 2010 in a ten-year-old commercial vineyard of own-rooted *Vitis vinifera* L. Cabernet Sauvignon in Penglai (37°48'N; 120°45'E), located in the east coast of Shandong Province, China. The experimental plot comprised fifteen 30-meter-length rows (40 vines per row) oriented north-south. The inter-rows had 2.5 m width. Vines were double-trunked, trained to a bilateral cordon at 0.8 m aboveground, spur-pruned annually, and covered with soil to assure overwinter protection. Shoots were trained upwards and each vine carried *ca.* 20 clusters with uniform management. Experimental rows were selected from these 12 center rows of the plot, and divided into six groups to create three rain-shelter treatment replicates and three open-field control replicates (two rows per replicate). All the experimental units were treated using similar production management practices.

The rain-shelter roof with a colorless polyethylene (PE) film was set up at 14th day after flowering (DAF, 28 June). For each row, rain shelter frame, with a height of 1.85 m from the ground, a width of 1.1 m, and an arc length of 1.25 m, was designed as a jackroof to eliminate the rain and provide the natural ventilation. The rows without the rain-shelter treatment were used as the control (also called open-field cultivation) in the same vineyard. At least one control row was left between the rain-shelter treated rows.

Sampling was carried out on the indicated dates. In this region, “Cabernet sauvignon” grape berries are harvested generally at about 120 DAF. In the present vintage, the grape berries started and completed coloring, and reached the technological harvest at 70 DAF, 98 DAF, and 126 DAF, respectively. To investigate whether the rain-shelter cultivation delayed the maturity of the grape berry, we harvested the grape berries by 140 DAF. Sampling time was fixed at 10:00–11:00 in the morning. The samples were placed in foam boxes and transported to the laboratory within two hours. Any physically injured, abnormal, or infected berries were removed. Each time, about 300 berries were collected from at least 100 clusters. Of these, 50 fresh berries were used for the analyses of soluble solid content and pH, whereas the rest of the samples were frozen in liquid nitrogen and stored at −80 °C. The berries were peeled and the skins were ground in liquid nitrogen to the fine powder, and then lyophilized for 24 h at −50 °C using an LGJ-10 vacuum freeze-dryer (Vibra-Schultheis, Offenbach am Main, Germany) prior to the analysis of anthocyanins.

### 3.2. Determination of Physicochemical Parameters

The soluble solids and pH value in the grape berries were determined according to a published method with minor modifications [27]. After squeezing the berries, the resultant juice was filtered through clean cheesecloth and decanted into a clean centrifuge tube. The filtrate was used for determination of the soluble solids and pH value. The percent soluble solids (Brix) was measured using a digital handheld pocket Brix refractometer (PAL-2, ATAGO, Tokyo, Japan), whereas pH value was analyzed using a pH meter (FE20, Mettler Toledo, Greifensee, Switzerland).

### 3.3. Extraction and Determination of Anthocyanins from Berry Skins

Anthocyanins in the grape skins were extracted according to the method described by Liang *et al.* [28]. Briefly, 0.5 g of the dry skin powder was extracted in methanol (10 mL) containing 2% formic acid. The extract was ultrasonicated for 10 min, followed by shaking in the dark for 30 min at 25 °C. Afterwards, the resultant mixture was centrifuged at 8000× *g* for 10 min and the supernatant was collected. The residues were re-extracted four more times using the same procedure. Finally, all the supernatants were pooled into a distilling flask. Methanol was removed using a rotary evaporator (SY-2000, Shanghai Yarong Biochemistry Factory, Shanghai, China) at 28 °C, and the residues were re-dissolved in 10 mL of 10% ethanol solution (pH 3.7). The extracts yielded were filtered through 0.45 μm filters (cellulose acetate and nitrocellulose, CAN) and directly used for HPLC analysis. The sample from a replicate was carried out in two independent extractions. As a result, each data point in the Tables and Figures represented the average of six values consisting of three biological replicates multiplied by two extraction replicates.

The analysis of anthocyanins was performed according to a previously published method [28] using an Agilent 1100 series LC-MSD trap VL instrument (Agilent Technologies, Santa Clara, CA, USA), equipped with a diode array detector (DAD) and a reverse-phase column (Kromasil C18, 250 × 4.6, 5 μm). The injection volume was 30 μL with a flow rate of 1.0 mL/min. The mobile phase was comprised of solvent A (water/acetonitrile/formic acid, 92:6:2, v/v) and solvent B (water/acetonitrile/formic acid, 44:54:2, v/v/v). The gradient elution was applied as follows: 10% B for 1 min, from 10% to 25% B for 17 min, isocratic 25% B for 2 min, from 25% to 40% B for 10 min, from 40% to 70% B for 5 min, from 70% to 100% B for 5 min. The column temperature was set at 50 °C and the detection wavelength on DAD was 525 nm. MS conditions were described as follows: Electro-spray ionization (ESI) interface, positive ion mode; Nebulizer pressure, 35 psi; dry gas flow rate, 10 mL/min; dry gas temperature, 350 °C, and mass scan mode, all mass scan from *m/z* 100–1000.

Five individual anthocyanins, including dephinidin-3-*O*-glucoside, petunidin-3-*O*-glucoside, malvidin-3-*O*-glucoside, cyanidin-3-*O*-glucoside, and peonidin-3-*O*-glucoside, were identified by comparing mass spectra and order of retention time with the commercially available standards. The identification of the remaining anthocyanins was achieved by analyzing the deprotonated ion and product ion of these compounds. The *cis* and *trans* isomers of the coumaroylates for peonidin-3-*O*-glucoside and malvidin-3-*O*-glucoside were distinguished by their elution time and concentrations. The *cis* isomer was generally eluted earlier on a reverse phase HPLC column and was present in a lower level compared to the *trans* isomer according to previously reported data [29,30,31,32,33,34,35].

### 3.4. Analysis of Transcript Level by Real-Time PCR

The total RNA of the grape berry skins was isolated using Universal Plant Total RNA Extraction Kit (Cat. # RP3301, BioTeke Co., Beijing, China), and digested with DNaseI (Code 2212, TaKaRa, Tokyo, Japan) to remove genomic DNA. The quality of RNA was verified by the existence of intact ribosomal bands following agarose gel electrophoresis and the absorbance ratios (A260/A280) of 1.8–2.0. The reverse transcription procedure followed a published method [36]. For cDNA synthesis, 2 μL of total RNA was reverse-transcribed in a 25 μL reaction mixture using M-MLV Reverse Transcriptase (M1707, Promega, Madison, WI, USA) and Oligo d(T)_18_ Primen (Code 3806, TaKaRa) with the manufacturer’s instruction. The synthesized cDNA was quantified and all the tested DNA samples were adjusted to the same concentration.

Relative expression of genes was measured by real-time PCR using SYBR^®^ *PremixEX* Taq^TM^ II (Code DRR081A, TaKaRa) on a 7300 Real Time PCR System (Applied Biosystems, Foster, CA, USA). PCR reaction mixture (20 μL) was comprised of 10 μL 2 × SYBR^®^*PremixEx* Taq^TM^, 8.5 μL ddH_2_O (TIANGEN, Beijing, China), 0.4 μL cDNA, 0.4 μL 50 × ROX Reference Dye II, and 0.7 μL primers (forward and reverse primers mixture, 10 μmol/L). The template cDNA was denatured at 95 °C for 30 s followed by 40 cycles of amplification at 95 °C for 10 s, 60 °C for 31 s, and a melt cycle from 60 °C to 95 °C. The sequences of the primers used for real-time PCR were referred from the previous studies [36,37,38] (Table 2). The specificity of the primers was verified by an agarose gel electrophoresis with one specific ribosomal band and dissociation curve with one specific peak. Quantification was normalized to *VvUbiquitin1* fragments amplified in the same conditions according to Bogs *et al.* [39]. There was identical amplification efficiency between target genes and internal reference gene. Each grape sample was conducted from three independent RNA extraction replicates to produce three RNA samples. Each RNA sample was performed from two technological replicates in analysis of real-time PCR.

**Table 2:** The primers of genes used in the Real-Time PCR assays.

Genes	Genbank Accession	Primer Sequences (5'–3')	Size of PCR Product (bp)
*VvF3'H*	AJ880357	F: CCAAGTTTTCGGGAAGTAAATG	171
		R: TACCCCTTGAGAATCATCGTTT	
*VvF3'5'H*	AJ880356	F: GCATGGATGCAGTTAAGTAGAAAA	113
		R: ATATGGCTTGGTGGTAGAATGAAACGA	
*VvUFGT*	AF000372	F: GGGATGGTAATGGCTGTGG	253
		R: ACATGGGTGGAGAGTGAGTT	
*VvUbiquitin*	BN000705	F: GTGGTATTATTGAGCCATCCTT	182
		R: AACCTCCAATCCAGTCATCTAC	

### 3.5. Statistical Analysis

Statistical analysis was performed by SPSS 11.5 software (Chicago, IL, USA). Duncun analysis was used to assess statistically significant differences in the content of various anthocyanins and the transcription levels of genes between the treated groups and the control. Sigma Plot 10.0 (Systat Software Inc., Richmond, CA, USA) was used to draft the graph. Each data point, expressed as milligram equivalent of the respective standard per kilogram of dried grape skin, was the average of three replications, *n* = 6.

## 4. Conclusions

Integrating the data from various analyses shown above, we found pattern changes between grape berry anthocyanins produced by rain-shelter cultivation and open-field cultivation. The life of green functional leaves was visibly prolonged under rain-shelter cultivation conditions. The rain-shelter cultivation did not alter the compositional proportion of anthocyanins, but increased the concentrations of almost all the anthocyanin components detected in the ripening berries. Correspondingly, the expression of *VvF3'H*, *VvF3'5'H* and *VvUFGT* was greatly up-regulated and this rising trend was kept until berry maturation. The accumulation period of both sugar and anthocyanins was prolonged by rain-shelter cultivation, which was strongly consistent with the delay of leaf senescence. In regions with grape growing season such as Penglai, the rain-shelter cultivation could increase wine quality of grape berries to a certain extent.
